# Risk of adverse pregnancy and infant outcomes associated with prenatal Zika virus infection: a post-epidemic cohort in Central-West Brazil

**DOI:** 10.1038/s41598-023-33334-5

**Published:** 2023-05-05

**Authors:** Luiza Emylce Pela Rosado, Celina M. Turchi Martelli, Elizabeth B. Brickley, Maria Barbara Franco Gomes, Talita de Toledo Lima, Paulo Sergio Sucasas da Costa, Marcos Pereira de Ávila, Marcello Braga Viggiano, Waldemar Naves do Amaral, Valeria Christina de Rezende Feres, Fabiola Souza Fiaccadori, Ana Laura de Sene Amancio Zara, Angela Ferreira-Lopes, Marilia Dalva Turchi

**Affiliations:** 1 Graduate Program in Tropical Medicine and Public Health of the Federal University of Goias, Goiânia, Brazil; 2 Obstetrics Department, Maternal and Infant Hospital of Goias State, Goiânia, Brazil; 3 Institute Aggeu Magalhaes, Fiocruz/Pernambuco, Recife, Brazil; 4 Department of Infectious Disease Epidemiology, London School of Hygiene & Tropical Medicine, London, United Kingdom; 5 Graduate Program in Health Sciences of the Federal University of Goias, Goiânia, Brazil; 6 Pediatric Department, Maternal and Infant Hospital of Goias State, Goiânia, Brazil; 7 Reference Center in Ophthalmology of the Federal University of Goias, Goiânia, Brazil; 8 Pediatrics Department, School of Medicine, Federal University of Goiás, Goiânia, Brazil; 9 Retina and Vitreous Department, School of Medicine, Federal University of Goias, Goiânia, Brazil; 10 Ultrasound Department, Maternal and Infant Hospital of Goias State, Goiânia, Brazil; 11 Obstetrics Department, School of Medicine, Federal University of Goias, Goiânia, Brazil; 12 Faculty of Pharmacy, Federal University of Goias, Goiânia, Brazil; 13 Virology Department, Institute of Tropical Pathology and Public Health, Federal University of Goias, Goiânia, Brazil; 14 Institute of Tropical Pathology and Public Health, Federal University of Goias, Goiânia, Brazil

**Keywords:** Infectious diseases, Paediatric research

## Abstract

This study aimed to estimate the risks of adverse infant outcomes in the first year of life related to prenatal Zika virus (ZIKV) exposure. A prospective cohort of pregnant women with rash was recruited in Central-West Brazil in a post-epidemic period (January 2017 to April 2019). We evaluated participants’ medical histories and performed ZIKV diagnostic testing using molecular (reverse transcription polymerase chain reaction [RT-PCR]) and serologic (immunoglobulin [Ig]M and plaque reduction neutralization tests [PRNT_90_]) assays. The ZIKV-positive group included both RT-PCR-confirmed cases as well as IgM and/or PRNT_90_-positive probable cases. Children were evaluated at birth and in the first 12 months of life. Transfontanellar ultrasound, central nervous system computed tomography, eye fundoscopy and retinography were performed. We estimated the absolute risk and 95% confidence interval (95% CI) of adverse infant outcomes among confirmed prenatally ZIKV-exposed children. Among 81 pregnant women with rash, 43 (53.1%) were ZIKV infected. The absolute risk of microcephaly among offspring of ZIKV-infected pregnant women was 7.0% (95% CI: 1.5–19.1), including the two cases of microcephaly detected prenatally and one detected postnatally. In total, 54.5% (95% CI: 39.8–68.7) of children in the ZIKV-exposed group had at least one ophthalmic abnormality, with the most frequent abnormalities being focal pigmentary mottling and chorioretinal atrophy or scarring. Our findings reinforce the importance of long-term monitoring of prenatally ZIKV-exposed children born apparently asymptomatic for Congenital Zika Syndrome.

## Introduction

The outbreaks of congenital microcephaly related to Zika virus (ZIKV) exposure during pregnancy, mainly identified in the Latin American and Caribbean region, led the World Health Organization (WHO) to declare a Public Health Emergency of International Concern in 2016^1,2^. Clinical, epidemiological and virological evidence have now firmly established the causal association between ZIKV infections during pregnancy and birth defects ^3–8^. Children who experience intrauterine ZIKV infections may subsequently present with a range of morphological and functional impairments collectively recognized as Congenital Zika Syndrome (CZS)^9–11^.

Several cohort studies, conducted mainly during the ZIKV epidemic period, have assessed the risk of adverse pregnancy outcomes associated with prenatal ZIKV exposure. A recent individual participant data meta-analysis of 13 Brazilian cohorts that followed up children with RT-PCR-confirmed prenatal ZIKV exposure reported absolute risks of microcephaly and abnormal neuroimaging findings of approximately 4% and 8%, respectively^12^. In Colombia, surveillance data of offspring with lab-confirmed prenatal ZIKV exposure reported the risk of ZIKV-associated birth defects to be approximately 9%^13^. In the French Territories in the Americas, a cohort study following up symptomatic RT-PCR-confirmed pregnancies reported risks of neurological and ocular defects possibly associated with ZIKV to be approximately 7% among exposed offspring^14^. In French Guiana, another cohort study of ZIKV infections during pregnancy reported a vertical transmission rate of approximately 26% and a risk of severe complications compatible with CZS to be 21% among ZIKV-positive fetuses/newborns^15^. In the United States territories and freely associated states, data from a ZIKV pregnancy registry estimated that approximately 14% of children with laboratory confirmed or possible prenatal ZIKV exposure presented with a birth defect and/or neurodevelopmental abnormality possibly associated with congenital ZIKV infection in the first two years of life^16^.

According to the Brazilian Public Health Event Registry (*Registro de Eventos em Saúde Pública,* RESP), the majority of the confirmed cases of Zika-related microcephaly were reported in the Northeastern region of the country. In this hotspot, the risk of microcephaly was 56.7 per 10,000 newborns during 2015–2016, the peak of the epidemic, compared with the historical risk of microcephaly of 2.0 per 10,000 newborns^17^. The Central-West region, where this study was conducted, was less affected with an estimated risk of microcephaly of 14.5 per 10,000 newborns during 2015–2016, with a sharp decline in subsequent years^17,18^. Using data from a prospective cohort of pregnant women who presented with rash (i.e., a common symptom of ZIKV infection) between January 2017 and April 2019 in the Goiânia metropolitan area in Central-West Brazil, this study aimed (i) to estimate the risks of adverse pregnancy and infant outcomes (i.e., in the first year of life) related to prenatal ZIKV exposure and (ii) to investigate whether specific symptoms could distinguish between ZIKV-positive and ZIKV-negative pregnant women with rash during a period of low ZIKV circulation. The current study represents, to our knowledge, the first evaluation of CZS risks in the Central-West region of Brazil.

## Results

**Figure 1:**
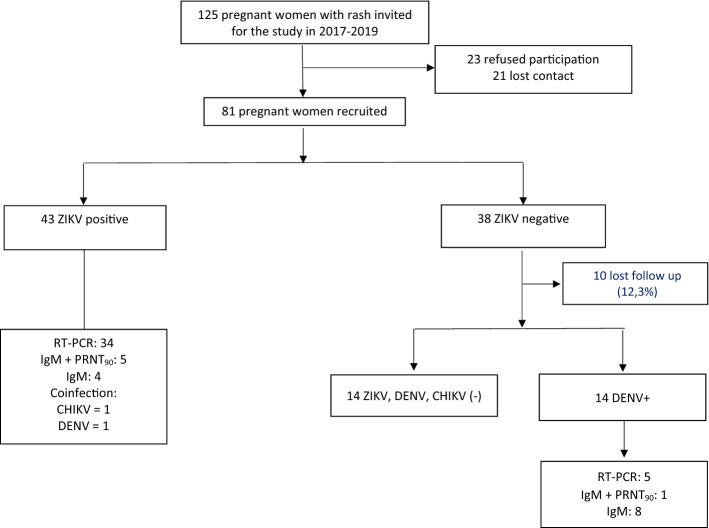
Flowchart of the recruitment and diagnosis of pregnant women with rash and suspected ZIKV infection from the cohort of Goiânia, Goiás, Brazil, 2017–2019.

Between January 2017 and April 2019, 125 pregnant women with rash and suspected ZIKV infection were identified in the city of Goiânia, Goiás, Brazil. Of the 81 who agreed to participate and were included in this cohort study, 43 (53.1%) women had laboratory-confirmed ZIKV infection based on molecular and/or serological tests. Of these 43 cases, 34 (79.1%) were confirmed positive for ZIKV maternal infection by RT-PCR, 5 (11.6%) by IgM and PRNT_90_, and four (9.3%) by IgM alone. Among the ZIKV-infected pregnant women, we detected one coinfection with chikungunya virus (CHIKV by RT-PCR) and one with dengue virus (DENV by PRNT_90_). Thirty-eight pregnant women tested negative for ZIKV, of whom 14 (36.8%) were DENV-positive by molecular or serological tests (Fig. 1).

Table 1 shows the sociodemographic characteristics of the enrolled pregnant women and the frequency of symptoms associated with the acute rash disease, according to laboratory results. The comparison was made between the group of ZIKV-positive pregnant women (n=43) versus the ZIKV-negative group (n=38). Most participants (n=79, 97.6%) were recruited during 2017–2018. In the ZIKV-positive group, 67.4% (n=29) of the pregnant women were recruited in 2017, and in contrast 89.4% (n=34) of the negative group were recruited in 2018. Only two cases were recruited in 2019 (up to April), and they were both ZIKV-negative. Among ZIKV-positive pregnant women, participants’ ages ranged from 16 to 39 years old (mean of 26.4), 75% reported having more than nine years of schooling, 63% had a family income higher than two times the Brazilian minimum wage per month, and 61% were of parda (*brown*). Overall, the frequency of acute exanthematic disease was distributed across pregnancy trimesters, with approximately one-third of cases in each trimester of pregnancy. The ZIKV-related rashes lasted for a median duration of four days and were accompanied mainly by pruritus, headache, prostration, arthralgia, myalgia, and fever. Other symptoms, including retro-orbital pain, photophobia, and upper respiratory symptoms, were reported less frequently. Conjunctival hyperemia was more common in the ZIKV-positive group (53.1%, n=10) versus the ZIKV-negative group (7.9%, n=3).

**Table 1:** Baseline characteristics of pregnant women with rash and suspected ZIKV infection from the cohort of Goiânia, Goiás, Brazil, 2017–2019.

**Variables**	Total N=81 (%)	Pregnant women	
		ZIKV (+) n=43 (%)	ZIKV (−) n=38 (%)
**Entry year in the cohort**			
2017	31/81 (38.3)	29/43 (67.4)	2/38 (5.3)
2018	48/81 (59.3)	14/43 (32.6)	34/38 (89.4)
2019	2/81 (2.4)		2/38 (5.3)
**Age (years)**			
Mean (SD)	26.4 (5.8)	25.8 (5.9)	27.0 (5.7)
**Schooling**			
≤ 9 years	20/81 (24.7)	8/43 (18.6)	12/38 (31.6)
> 9 years	61/81 (75.3)	35/43 (81.4)	26/38 (68.4)
**Family income**			
≤ 2 minimum wage	27/73 (37.0)	14/38 (36.8)	13/35 (37.1)
> 2 minimum wage	46/73 (63.0)	24/38 (63.2)	22/35 (62.9)
**Race/ethnicity**			
White	34/81 (42.0)	16/43 (37.2)	18/38 (47.4)
Black	5/81 (6.2)	1/43 (2.3)	4/38 (10.5)
Parda (Brown)	42/81 (51.9)	26/43 (60.5)	16/38 (42.1)
**Obstetrical background**			
First pregnancy	29/81 (35.8)	17/43 (39.5)	12/38 (31.6)
Previous abortion	12/40 (30.0)	6/22 (27.3)	6/18 (33.3)
Previous stillborn	4 (10.8)	2/21 (9.5)	2/16 (12.5)
**Type of pregnancy**^a^			
Single	68/71 (95.8)	40/43 (93.0)	28/28 (100.0)
Twin	3/71 (4.2)	3/43 (7.0)	0/28 (0)
**Trimester of ZIKV infection**			
1st trimester (≤ 13 weeks)	27/80 (33.7)	15/43 (34.9)	12/37 (32.4)
2nd trimester (> 13 to ≤ 28 weeks)	29/80 (36.3)	19/43 (44.2)	10/37 (27.0)
3rd trimester (≥ 29 weeks)	24/80 (30.0)	9/43 (20.9)	15/37 (40.6)
**Signs and symptoms of exanthematic disease**	N=72	n=39	n=33
Rash (days), median (IQR)	4 (3.0–7.0)	3 (3.0–6.0)	5 (3.5–7.0)
Pruritus	51/81 (63.0)	27/43 (62.9)	24/38 (63.2)
Headache	50/81 (61.7)	26/43 (60.5)	24/38 (63.2)
Prostration	42/81 (51.9)	25/43 (58.1)	17/38 (44.7)
Arthralgia	42/81 (51.9)	23/43 (53.6)	19/38 (50.0)
Myalgia	41/81 (50.6)	22/43 (51.2)	19/38 (50.0)
Fever	37/79 (46.8)	16/41 (39.0)	21/38 (55.3)
Retro-orbital pain	27/81 (33.3)	15/43 (34.9)	12/38 (31.6)
Photophobia	23/81 (28.4)	15/43 (34.9)	8/38 (21.1)
Upper respiratory	21/81 (25.9)	11/43 (25.6)	10/38 (26.3)
Conjunctival hyperemia	13/81 (16.0)	10/43 (53.1)	3/38 (7.9)

^a^Each pregnancy, including twin pregnancies, is considered as one. *SD* standard deviation, *IQR* interquartile range. Total numbers vary due to missing values.

We followed up 71 pregnant women and 74 children (i.e., from 68 singleton and three twin pregnancies). The three pairs of twins were in the maternal ZIKV-positive group. In total, 95.8% (n=68) of participants had serological screening data for syphilis, toxoplasmosis, cytomegalovirus (CMV), rubella, and HIV. All were HIV-negative and had no evidence of syphilis, rubella nor recent CMV infection. Two pregnant women had IgM-positive test results for toxoplasmosis, and both were in the ZIKV-negative group. Table 2 shows the characteristics of the prenatal follow-up until delivery. All pregnant women had at least two obstetric ultrasounds, and 76% (n=54) had three ultrasounds (one per trimester). Six cases of fetal abnormalities were detected by obstetric ultrasound.

**Table 2:** Characteristics of prenatal follow-up until delivery of women with rash and suspected ZIKV infection, Goiânia, Goiás, Brazil, 2017–2019.

Variables	Total N=71 (%)	Pregnant women	
		ZIKV (+) n=43 (%)	ZIKV (−) n=28 (%)
**Obstetrical ultrasound per trimester**			
1st trimester	62/71 (87.3)	36/43 (83.7)	26/28 (92.9)
2nd trimester	68/71 (95.8)	40/43 (93.0)	28/28 (100.0)
3rd trimester	66/71 (93.0)	40/43 (93.0)	26/28 (92.9)
**Abnormalities at obstetrical ultrasound**^a,b^			
2nd trimester	4/68 (5.9)	3/40 (7.5)	1/28 (3.6)
3rd trimester	6/66 (9.1)	5/40 (12.5)	1/26 (3.8)
**Obstetrical ultrasound findings**^a,b^			
Intrauterine growth restriction	6/66 (9.1)	5/40 (12.5)	1/26 (3.8)
Echogenic intracardiac structure	2/66 (3.0)	2/40 (5.0)	0/26 (0)
Cerebral calcifications, ventriculomegaly	2/66 (3.0)	2/40 (5.0)	0/26 (0)
Microcephaly	2/66 (3.0)	2/40 (5.0)	0/26 (0)
**Comorbidities/adverse events**^b^			
Hospitalization	8/61 (13.1)	3/36 (8.3)	5/25 (20.0)
Hypertension or pre-eclampsia	13/65 (20.0)	8/40 (20.0)	5/25 (20.0)
Urinary tract infection	10/57 (17.5)	8/34 (23.5)	2/23 (8.7)
Gestational diabetes	8/64 (12.5)	4/39 (10.3)	4/25 (16.0)
Premature rupture of membranes	2/55 (3.6)	1/35 (2.9)	1/20 (5.0)
Severe bleeding	2/65 (3.1)	2/41 (4.9)	0/24 (−)
**Type of labor**			
Transvaginal	26/68 (38.2)	17/42 (40.5)	9/26 (34.6)
Cesarean section	42/68 (61.8)	25/42 (59.5)	17/26 (65.4)

^a^Abnormalities might be in both trimesters. ^b^One or more complications are possible at the same woman and/or fetus. Numbers may vary due to missing values.

In this pregnancy cohort, there were no miscarriages or stillbirths. During the gestational period, 13.1% of women required hospitalization. Overall, the most frequent comorbidities during pregnancy were hypertension (20.0%) with one eclampsia case, urinary tract infection (17.5%) and gestational diabetes (12.5%), with no statistical difference between groups. Premature delivery occurred in 14.3% of the ZIKV-positive group versus 3.7% of the ZIKV-negative group. There were similar frequencies of labor induction and elective delivery between the ZIKV-positive and ZIKV-negative groups. Cesarean section (61.8%) was the most common delivery route in both groups (Table 2).

Overall, 72 newborns, of whom 45 were children in the maternal ZIKV-positive group and 27 were in the ZIKV-negative group, were evaluated from delivery up to one year of age (Table 3). Two pregnant women, one from each group, were lost to follow-up just before labor. The median values for APGAR scores in the 5th minute, weight, length at birth and head circumferences were similar between the groups. Four neonates with microcephaly were detected at birth, all in the ZIKV-exposed group.

**Table 3:** Pediatric assessment of newborns and infants born to ZIKV-exposed and unexposed pregnant women of the cohort from Goiânia, Goiás, Brazil, 2017–2019.

Variables	Total N=72 (%)	Groups		*p* value
		ZIKV (+) n=45 (%)	ZIKV (−) n=27 (%)	
**Male**	38/72 (52.8)	23/45 (51.1)	15/27 (55.6)	0.715^a^
**Gestational age (weeks)**				
Median (IQR)	39 (38–40)	39 (37.5–40)	39 (38–40)	0.375^b^
Minimum-maximum	30–42	30–42	36–41	
Preterm birth (weeks)	9/72 (12.5)	8/45 (17.7)	1/27 (3.7)	0.140^c^
Very preterm (≥28 to <32)	2/72 (2.8)	2/45 (4.4)	0/27 (0)	0.524
Moderate to late preterm (≥32 to <37)	7/72 (9.7)	6/45 (13.3)	1/27 (3.7)	0.244
**5th minute APGAR**	N=67	n=44	n=23	
Median (IQR)	9 (9–10)	9 (9–10)	10 (9–10)	0.146^b^
**Birth weight (grams)**	N=70	n=44	n=26	
Median (IQR)	3162 (2800–3552)	3150 (2760–3466)	3428 (2867–3607)	0.126^b^
**Length at birth (centimeters)**	N=68	n=43	n=25	
Median (IQR)	50 (48–51)	49 (47–51)	50 (48–51)	0.292^b^
**Head circumference (centimeters)**	N=70	n=44	n=26	
Median (IQR)	34.5 (33–35)	34 (33–35)	35 (34–35)	0.469^b^
Minimum-maximum	25–37	25–37	33–37	
**Small for gestational age**	6/70 (8.6)	5/44 (11.4)	1/26 (3.8)	0.538^c^
**Microcephaly at birth**	4/70 (5.7)	4/44 (9.1)	0/26 (0)	0.167^c^
Moderate (> −3 to ≤ −2 SD)	3/70 (4.3)	3/44 (6.8)	0/26 (0)	0.289
Severe (≤ −3 SD)	1/70 (1.4)	1/44 (2.3)	0/26 (0)	1.000
**Transfontanellar ultrasound and/or computed tomography exams**	37/70 (52.8)	29/44 (65.9)	8/26 (30.7)	0.004
Any abnormality	4/37 (10.8)	4/29 (13.8)	0/8 (0)	0.495^c^
Calcifications	4/37 (10.8)	4/29 (13.8)	0/8 (0)	0.495^c^
Ventriculomegaly	3/37 (8.1)	3/29 (10.3)	0/8 (0)	0.666^c^
Focal or diffuse cortical atrophy	3/37 (8.1)	3/29 (10.3)	0/8 (0)	0.666^c^
**Pediatric evaluation**	59/72 (81.9)	43/45 (95.6)	16/27 (59.3)	0.002
Postnatal microcephaly^d^	3/59 (5.1)	3/43 (7.0)	0/16 (0)	0.561^c^
**Ophthalmological evaluation**	55/72 (76.4)	44/45 (97.8)	11/27 (40.7)	<0.001
Any eye anomaly	26/55 (47.3)	24/44 (54.5)	2/11 (18.6)	0.060^c^
Optic nerve anomaly^e^	7/55 (12.8)	7/44 (15.9)	0/11 (0)	0.261^c^
Hypoplasia	4/55 (7.3)	4/44 (9.1)	0/11 (0)	0.573
Pallor	2/55 (3.6)	2/44 (4.5)	0/11 (0)	1.000
Excavation	3/55 (5.4)	3/44 (6.8)	0/11 (0)	1.000
Retinal anomaly^e^	25/54 (46.3)	23/43 (53.5)	2/11 (18.2)	0.046^c^
Chorioretinal atrophy	8/54 (14.8)	8/43 (18.6)	0/11 (0)	0.184
Chorioretinal scarring	3/54 (5.6)	3/43 (7.0)	0/11 (0)	1.000
Focal pigmentary mottling	12/54 (22.2)	12/43 (27.9)	0/11 (0)	0.041
Retinal hemorrhage	3/54 (5.6)	3/43 (7.0)	0/11 (0)	1.000
Retinal vascular tortuosity	1/54 (1.9)	1/43 (2.3)	0/11 (0)	1.000
Peripheral pigment dispersion	7/54 (13.0)	5/43 (11.6)	2/11 (18.2)	0.621
**Neurological assessment**	47/72 (65.3)	34/45 (75.6)	13/27 (48.1)	0.018
Disorders in clinical evaluation	6/47 (12.7)	4/34 (11.8)	2/13 (15.4)	0.999^c^
Seizures and/or neurologic impairment^f^	3/47 (6.4)	3/34 (8.8)	0/13 (0)	0.550
Motor deficit and/or disturb in tone or trophism	3/47 (6.4)	1/34 (2.9)	2/13 (15.4)	0.181

a. Chi-squared test. b. Mann-Whitney U test. c. Fisher’s exact test. d. 2 of the 3 postnatal microcephaly cases where detected at birth. e. One or more anomalies are possible in the same child. f. Neurologic impairments include: change in tone or trophism, hyperexcitability, localized motor deficit, behavior change, and signs of pyramidal release. Numbers may vary due to missing values. *IQR* Interquartile range, *SD* Standard deviation.

In addition, morphological malformations in newborns and children were investigated by clinical evaluation (pediatrician, ophthalmologist, and neurologist) and complementary exams. In total, the mean number of clinical evaluations per child was 3.1 (standard deviation, SD: 1.1) appointments. Children with maternal ZIKV exposure had significantly greater chances of follow-up than unexposed children in the first year of life (Table 3).

All children with microcephaly and/or brain imaging abnormalities were in the ZIKV-exposed group. Four infants had microcephaly detected at birth. Of these, two microcephalic infants had ZIKV exposure in the 1st trimester, prenatal morphological alterations confirmed by transfontanellar ultrasound (i.e., unilateral ventriculomegaly, periventricular calcifications, and cortical atrophy), and were classified as having postpartum disproportional microcephaly. Additionally, one of the two children with disproportional microcephaly was classified as having severe microcephaly, occipital bulge, calcification of the cranial sutures, and arthrogryposis. Both children with disproportional microcephaly had severely impaired neurodevelopment during medical follow-up and fulfilled the criteria for severe complications compatible with CZS. The other two cases, classified as having mild proportional microcephaly at birth, had normal postpartum growth and development over the first year of life. Both fetuses had intrauterine growth restriction and normal neuroimaging results at prenatal ultrasound. One child with proportional microcephaly presented with chorioretinal atrophy and focal pigmentary mottling and was classified as having mild/moderate signs potentially associated with CZS. The other child with proportional microcephaly presented with chorioretinal atrophy as the only abnormality detected. Supplementary Table (S1) presents the main clinical characteristics, prenatal and postnatal imaging, and ZIKV RT-PCR results for all six children with brain abnormalities potentially associated with CZS.

Among the ZIKV-exposed group, two children, born with normal head circumferences, presented with neurological abnormalities by transfontanellar ultrasound. One child, with linear perithalamic calcifications, maintained a normal head circumference but presented with neurological delays during follow-up and was classified as having mild/moderate signs potentially associated with CZS, without microcephaly. The other child had frontoparietal calcifications, bilateral ventriculomegaly, and developed severe postnatal microcephaly, abnormal neurological development, and was classified as having severe complications compatible with ZIKV. Of note, two children in the unexposed group also had mild abnormalities at the neurological assessment at three months of age; one had abnormal tone/trophism, while the other had inadequate visual response and mild motor deficit. Both had normal development upon subsequent clinical evaluations.

**Figure 2:**
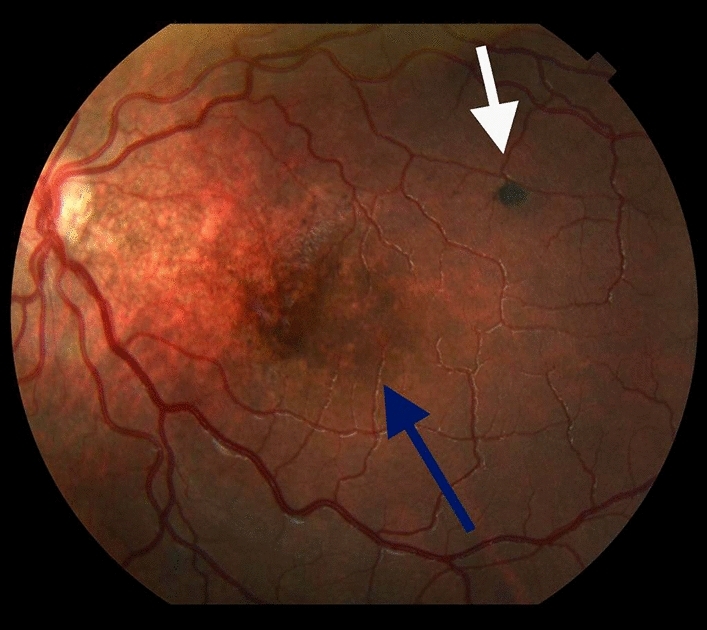
Color retinography of a preterm infant exposed to ZIKV with focal pigmentary mottling (blue arrow) with chorioretinal scarring (white arrow).

**Figure 3:**
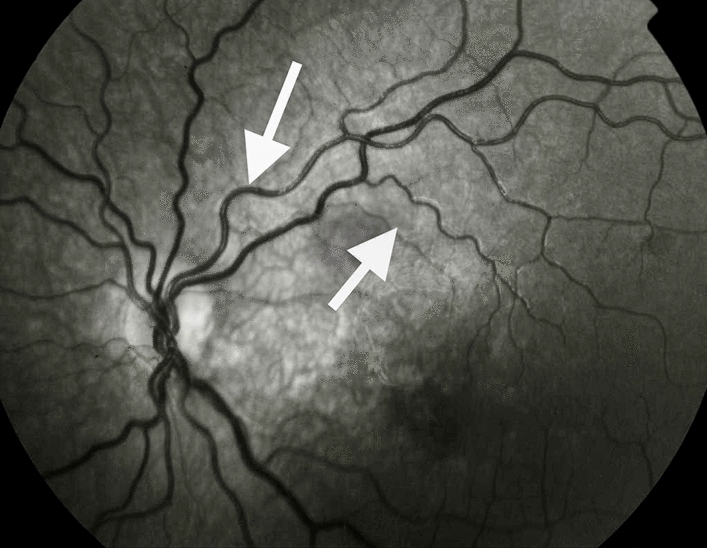
Monochrome retinography of the same case showing retinopathy of prematurity without retinal hemorrhage. The white arrows show the typical tortuous vessels of retinopathy of prematurity.

Most children (76.4%, n=72) had ophthalmological evaluations (i.e., funduscopic examination and/or retinography scan). Of the evaluated children, 26 out of 55 children (47.3%) had at least one ophthalmic abnormality. Ophthalmic abnormalities were detected in 54.5% (n=24) of ZIKV-exposed children versus 18.2% (n=2) in ZIKV-unexposed children (*p*=0.06) (Table 3). The most common ophthalmological findings among ZIKV children were: focal pigmentary mottling (12/24, 50.0%), chorioretinal atrophy or chorioretinal scarring (11/24, 45.8%), and optic nerve hypoplasia with or without pallor and excavation (7/24, 29.2%). More details on ophthalmological findings among ZIKV-exposed infants with and without microcephaly and/or neuroimaging abnormalities can be found in Supplemental Tables S1 and S2. In addition to having retinopathy of prematurity (ROP), three preterm infants presented with focal pigmentary mottling; of these, one also had chorioretinal atrophy. Figure 2 depicts color retinography of a preterm infant with focal pigmentary mottling with chorioretinal scarring. Figure 3 shows retinal hemorrhage and typical tortuous vessels of prematurity in the same case as Fig. 2.

Overall, 10 out of 61 (16.4%) children had minor heart abnormalities detected on transthoracic echocardiogram (ECHO) during the neonatal period. Of these, seven cases had patent foramen ovale (PFO), two had ostium secundum septal defect, and one had tricuspid physiological regurgitation. Four of the children with heart abnormalities were premature, with gestational ages ranging from 30.4 to 36.6 (weeks.days). Minor heart defects were detected in 21.6% (8/37) of the ZIKV-exposed group and in 8.3% (2/24) of the unexposed group. Of note, one child with microcephaly had echogenic intracardiac structure (golf ball) at prenatal ultrasound and also 3 mm PFO on ECHO. Overall, no severe cardiac defects were detected.

Table 4 presents the main adverse outcomes among the ZIKV-exposed fetuses and children. Considering the obstetrical ultrasound findings 5.0% (95% CI: 0.9–15.5) of fetuses presented with microcephaly, cerebral calcifications and ventriculomegaly prenatally. The absolute risk of microcephaly at birth was 9.1% (95% CI: 3.7–17.9). The risk of microcephaly within one year of age was 7.0% (95% CI: 1.5–19.1) including two cases detected prenatally and one detected postnatally. These three children with microcephaly met the criteria for severe complications compatible with CZS. Two children without microcephaly presented with signs potentially associated with mild/moderate CZS. Therefore, the absolute risk of mild/moderate to severe CZS was 11.4% (95% CI: 4.3–23.4). In total, 54.5% (95% CI: 39.8–68.7) of ZIKV-exposed children presented at least one ophthalmological anomalies (retinal and/or optic nerve).

**Table 4:** Main adverse outcomes among fetuses and infants vertically ZIKV-exposed from the cohort of Goiânia, Goiás, Brazil, 2017-2019.

**Outcomes**	ZIKV-exposed group n=44	% (95% CI)^a^
**Primary outcomes**		
Microcephaly within one year of age	3/43	7.0% (1.5–19.1)
Congenital Zika Syndrome	5/44	11.4% (4.3–23.4)
**Secondary outcomes**		
Microcephaly by prenatal ultrasound or abnormal brain imaging	2/40	5.0% (0.9–15.5)
Microcephaly at birth	4/44	9.1% (3.7–17.9)
Ophthalmological anomalies (retinal and/or optic nerve)	24/44	54.5% (39.8-68.7)

^a^95% Confidence interval.

## Discussion

Approximately two years after the peak of the ZIKV epidemic in Brazil, we detected three microcephaly cases (7.0%) among a cohort of ZIKV-positive pregnant women in the Central-West region of Brazil. In our study, most participants were recruited in 2017 and 2018. Whereas 67% of the ZIKV-positive women were detected in 2017, 89% of the ZIKV-negative group were recruited in 2018. Consistent with previous findings from an ecological study using surveillance data from Goiânia^18^, our findings underscore the persistence of the ZIKV circulation beyond the peak in 2015–2016 but also highlight the rapid decline of ZIKV transmission in subsequent years. These observations are also consistent with national surveillance data^19,20^ as well as the broader epidemiological trends in ZIKV circulation across the American region^21^.

In our cohort of 81 pregnant women with rash, 43 were laboratory confirmed to have ZIKV. In addition, DENV was detected in 50% of the ZIKV-negative group, showing the cocirculation of ZIKV and DENV in our study setting. These findings are consistent with the Brazilian surveillance system and other field studies conducted in urban areas with high populations of *Aedes* spp. mosquitoes^22,23^. At baseline, pregnant women with rash had similar clinical presentations regardless of whether they were positive for ZIKV, DENV, or CHIKV, as described in other Brazilian regions^24–27^. Due to the similarity of clinical manifestations, short viremia duration, and the serological cross-reactivity between flaviviruses (i.e., DENV and ZIKV), it is urgent to have highly specific point of care molecular tests for rapidly differentiating between DENV, ZIKV, and CHIKV in endemic regions. We also identified two ZIKV infected pregnant women simultaneously infected with CHIKV or DENV, but they did not present with more severe clinical presentations. These findings align with a recent systematic review that provided evidence that ZIKV coinfections, mainly with CHIKV or DENV, do not appear to influence the clinical manifestation of uncomplicated ZIKV infections in adults in general^28^.

In the present study, prenatal ultrasound exams detected two fetuses with microcephaly, cerebral calcifications, and ventriculomegaly diagnosed as CZS. Abnormal prenatal ultrasonography results were well documented in a cohort of 92 ZIKV-infected women during the emergence of ZIKV in Rio de Janeiro, Brazil^3^. However, normal ultrasound results do not exclude the risk of adverse outcomes at birth^29^. In Brazil, reports of CZS were mainly diagnosed in the postnatal period, and only 2% of the cases were identified by prenatal ultrasound^30^, suggesting potential underreporting of congenital anomalies in the official surveillance system.

Our result of an 11.4% risk of CZS is consistent with the 12.7% risk of severe adverse and neonatal outcomes reported in a cohort of pregnant women residing in French Guiana that was conducted in the epidemic and post-epidemic period^31^. However, our finding was higher than the 3.9% (95% CI: 2.5–5.9) risk of CZS, microcephaly and/or other central nervous system malformations reported among live births during the peak of the epidemic in Southeast of Brazil^32^. These discrepancies are likely explained, in part, by differences in the length of follow-up as well as the performance of in-depth eye examinations (e.g., retinography).

In our cohort, the estimated absolute risk of microcephaly among children born to ZIKV-infected pregnant women was 7.0% (95% CI: 1.5–19.1), including the two cases detected prenatally and one case with microcephaly detected postnatally. Of note, all three children with Zika-related microcephaly were born to pregnant women with rash reported in the first trimester of the gestational period. This finding is in line with other studies that have estimated the risk of microcephaly and/or other major signs of CZS among offspring of pregnant women with ZIKV infection to vary from 1.9 to 4.5%^3,24,29,32,33^. One question raised in the early ZIKV pandemic was whether the absolute risk of Zika-related microcephaly varied according to the geographic setting^34^. A recent meta-analysis of 13 Brazilian cohorts of ZIKV infected women showed strong evidence that the risk of microcephaly was relatively homogeneous across the regions^12^. Therefore, the variation of risk among the different cohort studies may be due to study design-related discrepancies, such as the different criteria of maternal exposure and the range of short and long-term outcomes evaluated^35^.

Regarding the broad spectrum of congenital ZIKV infection, one of the key findings from our research is the high prevalence (~55%) of eye defects detected in ZIKV-exposed children with or without brain compromise. This high frequency of ocular abnormalities may be due to the early fundoscopic eye exam that identified patients to undergo retinography. The retinography offers a good posterior pole detail, allowing the early detection of subtle anomalies such as focal pigmentary mottling.

In a cohort conducted in Rio de Janeiro, 21.4% of prenatally ZIKV-exposed infants had eye abnormalities^36^, primarily in the absence of other neurological abnormalities. Other studies have reported a high frequency of eye abnormalities ranging from 21.4% to 70.0% among children with CZS, with optic nerve and retinal abnormalities as the most common findings^37,38^. This large variation may be due to differences in participants` inclusion criteria, diagnostic tools, and follow-up time. Although uncertainty remains regarding the clinical significance of minor eye anomalies in infants without other manifestations of CZS, prolonged monitoring of these children and their vision remains important.

In our cohort, approximately one-fourth of the ZIKV-exposed group had minor cardiac findings, mainly persistent foramen ovale, detected on ECHO during the neonatal period. Persistent foramen ovale is a common asymptomatic defect expected to resolve spontaneously with increasing age. Other studies have also reported high frequencies of minor/mild heart defects noted on ECHO among ZIKV-exposed infants^39–41^ that are higher among ZIKV-exposed infants than expected for the population^42^. This higher prevalence of heart defects among prenatally ZIKV-exposed children may be explained by the use of a sensitive screening method (ECHO) to investigate heart defects in asymptomatic neonates. So far, there is no evidence of the association between ZIKV infection and congenital heart defects. Currently, heart defects are not considered adverse outcomes of congenital ZIKV infection in position papers and guidelines^16,43–45^.

The strengths of our study consist of its prospective design using a standardised protocol in collaboration with the Microcephaly Epidemic Research Group (MERG, Brazil) as part of the ZikaPLAN consortium^46,47^. In addition, laboratory evidence of recent maternal ZIKV exposure by molecular and serological tests were performed in a reference laboratory. A team of specialists investigated fetal and postnatal adverse outcomes up to the first year of life. One limitation of the study is that not all neonates/infants were tested for ZIKV infection due to difficulty in collecting specimens from apparently healthy infants without clinical signs/symptoms. Another limitation is that the children prenatally exposed to ZIKV had higher adherence to scheduled medical visits compared to the ZIKV-negative group. While a possible follow-up bias cannot be excluded, it does not affect the estimated absolute risks among the ZIKV-exposed group. This cohort is also limited by its small sample size. Nevertheless, our study represents approximately 90% of the pregnant women with rash and confirmed ZIKV infection notified to the local surveillance system in the study period. The small number of pregnant women with rash registered in the post-outbreak period in Goiânia hampered our ability to estimate less frequent adverse pregnancy outcomes, such as stillbirth. Also, this type of passive surveillance that excludes the asymptomatic cases clearly underestimates the entire ZIKV-positive women population in the local community. Implementation of prenatal ZIKV screening during non-epidemic years remains under discussion^48^.

Our study highlighted the relevance of prenatal ultrasound findings in the early diagnosis of CZS. Further, our research underscores the urgent need for point of care molecular tests to distinguish arbovirus infections due to the overlap of clinical signs, the short viremia period, and the serological cross-reactivity among flaviviruses. Our analysis, particularly our ophthalmological findings, also reinforces the importance of long-term follow-up of prenatally ZIKV-exposed children born apparently asymptomatic for CZS. Finally, congenital ZIKV infection continues to affect neonates in the post-epidemic period, and it is a persistent threat in endemic areas, such as Brazil. Vaccines to prevent maternal ZIKV infections are urgently needed in regions with *Aedes* spp. mosquitoes.

## Methods

The project was approved by the Research Ethics Committee of the Federal University of Goiás, Brazil (Ethical Clearance Certificate—CAAES: 64534017.7.0000.5083 and 64534017.7.3001.5080). We confirm that all methods were performed in accordance with the Declaration of Helsinki and Brazilian Resolution (MS/CNS 466/2012) and regulations regarding research on human subjects.

### Study design

This investigation is a prospective cohort study with follow-up from January 2017 to April 2019 in pregnant women who presented with rash and resided in the metropolitan area of Goiânia in Goiás state in the Central-West of Brazil. Goiânia has an estimated population of 2.5 million inhabitants, encompassing 20 municipalities, and approximately 38,200 live births per year^49^. Since February 2016, suspected cases of ZIKV infection in pregnant women have been reported to the national Notifiable Diseases Surveillance System (*Sistema de Informação de Agravos de Notificação*, SINAN)^50^. In early 2017, we established a prenatal clinic for exanthematic diseases at the public referral hospital for high-risk pregnancies and pediatric care (Hospital Estadual Materno Infantil Dr. Jurandir do Nascimento, HMI), where this cohort was based.

Potentially eligible participants for this study were identified (i) from records in the compulsory SINAN notification system for ZIKV infections during pregnancy or (ii) from those seeking attendance at the emergency ward and referred to the prenatal clinic for exanthematic diseases at HMI and, then, were invited to participate in the study by phone call or personal contact by the research team fieldworkers.

### Participants of this cohort study

Pregnant women over 15 years of age who presented with a macular or papular rash and two or more other symptoms, including fever, arthralgia, headache, myalgia, or non-purulent conjunctivitis, in accordance to definition of suspected ZIKV cases adopted by the Brazilian Ministry of Health^43^. No exclusion criteria were applied.

After signing the Free and Informed Consent Form, women answered a structured questionnaire regarding sociodemographic, clinical-epidemiological, and reproductive characteristics. We used standardised protocols and forms developed by the Microcephaly Epidemic Research Group (MERG) from Pernambuco, Brazil, to collect data^24^. We investigated current and past medical and obstetric conditions. The pregnant women were asked about the characteristics of the rash and associated signs and symptoms, in addition to potential maternal comorbidities that could affect fetal growth and/or development. The interviews were conducted by trained health professionals from our research team.

### Laboratory tests

As part of the public health surveillance during the study period, pregnant women with suspected ZIKV infections who attended public health units in the Goiânia metropolitan region were invited to provide blood and/or urine samples for confirmatory tests, which were performed at the Central Laboratory of Public Health Dr. Giovanni Cysneiros in Goiás State (LACEN-GO). Molecular testing with reverse transcription—polymerase chain reaction (RT-PCR) was performed within the first five days of symptom onset for blood samples and up to 10 days after symptom onset for urine samples. Serology was performed for blood samples collected after the 5th day of symptom onset to detect immunoglobulin (Ig)M antibodies to ZIKV and DENV^51^.

At baseline interview, pregnant women provided blood and urine samples for additional molecular (ZIKV and chikungunya virus- CHIKV) and serological tests (ZIKV, DENV, and CHIKV). Samples were sent to the Laboratory of Virology and Cell Culture (LabVICC) of the Institute of Tropical Pathology and Public Health, and to the Laboratory of Molecular Biology (BioTec) of the Pharmacy Faculty of the Universidade Federal de Goiás (UFG). For infants with brain abnormalities with or without microcephaly, we collected blood and/or urine samples for RT-PCR ZIKV detection of ZIKV.

For the RT-PCR assays, the viral RNA was extracted from 140 µL of serum samples using the QIAamp® Viral RNA Mini Kit (Qiagen, Germany), following the manufacturer's instructions. The RT-PCR assays used probes and primers previously described for ZIKV^52^ and CHIKV^53^. The reaction system and amplification conditions were performed according to the manufacturer's specifications in the commercial Path-ID multiplex kit One-Step RT-qPCR 1X (Applied Biosystems—Carlsbad, CA) in a Rotor-Gene Q Real-time PCR thermocycler (Qiagen, USA). The result was considered positive when the cycle threshold (Ct) value was ≤38.

Serological tests were performed using commercially available diagnostic Enzyme Linked Immunosorbent Assay (ELISA) kits for the detection of IgM antibodies to ZIKV (Euroimmun® Lübeck, Germany), DENV (DXSelectTM Focus Diagnostics Inc., USA), and CHIKV (Euroimmun®, Lübeck, Germany). Mother´s samples were tested using the US Centers for Disease Control and Prevention IgM antibody capture (MAC)-ELISA ‘in house’ protocol. To confirm ZIKV infection, serum samples with an inconclusive or seropositive result were tested for the presence of anti-ZIKV neutralizing antibodies by PRNT_90_^54^. Briefly, serum samples were diluted and mixed with a viral suspension (ZIKV, DENV−1 to 4). After incubation, the virus-antibody mixture was added in a confluent monolayer of Vero cells (100 plaque forming units, PFU) and covered with carboxymethyl cellulose. The cut-off value was defined based on a 90% of plaque reduction (PRNT_90_) at the lowest dilution tested (1:20). Serum samples were considered positive when antibody titers were ≥1:20^55^.

### Exposure definition

Exposure definition was based on a combination of molecular, serologic and plaque reduction neutralization tests for ZIKV. Pregnant women with suspected ZIKV infections were categorized into three groups. They were considered to have (i) confirmed maternal ZIKV infection if the pregnant women tested ZIKV-positive by RT-PCR; (ii) probable maternal ZIKV infection if the pregnant women had at least one ZIKV-positive IgM result, with or without a ZIKV-positive plaque reduction neutralization test (PRNT_90_); or (iii) ZIKV-negative if all tests (i.e., RT-PCR, IgM, or PRNT_90_) for recent infection yielded negative results. Confirmed and probable cases of ZIKV infection among pregnant women were considered as maternal ZIKV-positive group.

### Pregnancy follow-up

Pregnant women were followed-up every four to eight weeks until delivery by the project team, independent of their routine prenatal care. All follow-up visits (consultations) were carried out with the assistance of a single obstetrician (LEPR) for consistency and quality assurance. Spontaneous fetal loss before the 22nd week of pregnancy was defined as miscarriage. A fetal loss after this period was defined as stillbirth. The birth of a live newborn before the 37th week of pregnancy was considered a premature birth.

### Pediatric follow-up and definitions

Newborn data, including anthropometric measurements, morphological and functional status were extracted from medical records. Children were referred to specialized health care units (Hospital Materno Infantil, Hospital das Clinicas and Centro de Referencia em Oftalmologia, CEROF da UFG). They were evaluated by neonatologists, pediatricians, pediatric neurologists and pediatric ophthalmologists, of the research team using standardised forms and protocols. Pediatric visits were scheduled as early as possible in the neonatal period and then at 3, 6, and 12 months of life. Children were monitored as clinically indicated with additional medical visits.

Newborns weighing less than 2500g at birth were considered low birth weight, and small- for-gestational-age (SGA) was defined as a birth weight less than the 10th percentile for gestational age and sex based on the INTERGROWTH-21st curves^56,57^.

Microcephaly was defined as head circumference (HC) (i.e., measured at birth and confirmed by physical examination between 24 and 48 hours afterward) below two standard deviations for gestational age and sex of the reference values of the INTERGROWTH-21st curves for boys and girls. Disproportionate microcephaly was defined as a HC z-score of < −2 with a normal birth weight score of > −2^56^. Postnatal-onset microcephaly was defined for children with no evidence of microcephaly at birth but who had HC measurements collected >2 weeks after birth, that fell below the 3rd percentile of the curve for the child’s sex and age based on the World Health Organization child growth standards^58^.

Ophthalmological evaluation was performed by two ophthalmologists (at least one of them a retinal specialist) and image results were reviewed by two other specialists blinded to the mothers’ ZIKV infection status. Ophthalmoscopy was performed with a Riester All Pupil II—Xenon indirect binocular ophthalmoscope (Rudolf Riester GmbH, Germany) and a non-contact lens (Double Aspherics) of 28 diopters (Volk, United States). Photo documentation was performed with a Topcon TRC-50IX Retinal Camera® (Topcon Medical Systems) (TOPCON CORPORATION, 2008) or Visucam Fundus Imaging 524® (Carl Zeiss Meditec Group) (ZEISS, 2017).

For children with clinically presumed heart defects transthoracic echocardiograms (ECHO) with standard pediatric views were performed at the neonatal period. Minor ECHO abnormalities were defined as persistent foramen ovale (PFO), small physiological tricuspid regurgitation, physiological pulmonary stenosis and patent ductus arteriosus in premature infants. Other heart defects were classified as major. Severe birth defects were defined as the ones requiring therapeutic interventions in the first days of life^39,59^.

We considered microcephaly, brain imaging abnormalities, neurological and/or ophthalmologic abnormalities as signs and symptoms potentially associated with CZS according to the Brazilian guidelines^43^. Congenital Zika Syndrome with severity categorized based on a combination of major and minor signs (neuroimaging exams, clinical signs and ocular anomalies) proposed by Pomar and colleagues^15,44^ We considered as neurological or neurodevelopmental abnormalities: seizures, developmental delay, cognitive disabilities, localized motor deficit, hypertonia, alterations in tone or trophism, signs of pyramidal release, swallowing disorders, and movement disorders^16^.

### Pre- and postnatal ultrasound exams

To determine the gestational age and subsequently to identify fetal anomalies, at least two ultrasounds were performed, one at the time of recruitment and one in the third trimester of pregnancy, between the 32nd and 37th gestational weeks. For the confirmation of gestational age, a first ultrasound performed in the first 12 weeks of gestation was preferred, which was compared to the date of the last menstruation provided by the pregnant woman. Whenever possible, fetal morphological ultrasound was also requested between 22 and 26 weeks of gestation^60^.

Antenatal ultrasound exams done at the referral hospital (Hospital Materno Infantil) were performed by a single examiner (fetal medicine specialist), with a 3.5 MHz convex transducer (Philips Healthcare device, model HD7), to measure the fetal head circumference (HC), other biometric parameters and estimate fetal weight. The diagnosis of intrauterine microcephaly was performed when the HC measurement was less than 2 standard deviations from the lower limit of the normality curve for the respective gestational age^61,62^.

Transfontanellar ultrasound was performed at the availability at the maternity hospital where the delivery took place and later on until the first six months of life, to investigate Central Nervous System abnormalities. Qualified physicians performed imaging examination, following a protocol for evaluating and describing results.

### Fetuses and infants outcomes

Primary outcomes included: (i) microcephaly within one year of age and (ii) Congenital Zika Syndrome. The secondary outcomes were: (i) fetal loss, (ii) prenatal microcephaly detected by ultrasound, (iii) microcephaly at birth, and (iv) ophthalmological anomalies (retinal and/or optic nerve).

### Data processing and statistical analysis

All data collected were stored using Research Electronic Data Capture (REDCap). After assessing the consistency of the data, statistical analyses were performed using IBM SPSS Statistics 25 version. For continuous variables with normal distribution, we used the student’s t-test. Otherwise, the Mann-Whitney U test was used to compare these variables. We applied the Chi-squared test or Fisher’s exact tests for categorical variables. *p* values of less than 0.05 were considered statistically significant. We estimated the absolute risk and 95% confidence interval (95% CI) for primary and secondary adverse outcomes among ZIKV-exposed children.

## Supplementary Information

Supplementary Information 1.

Supplementary Information 2.

## Data Availability

The datasets generated during and analysed during the current study are available from the corresponding author on reasonable request.
