# Supplementary material for: Risk of adverse pregnancy and infant outcomes associated with prenatal Zika virus infection: a post-epidemic cohort in Central-West Brazil
Source: Sci Rep. 2023 May 5;13:7335. doi: 10.1038/s41598-023-33334-5 (PMC10161159; doi:10.1038/s41598-023-33334-5)
Supplement: Supplementary file 1 — Supplementary Information 1. [file 41598_2023_33334_MOESM1_ESM.docx]

Supplementary Table 1. Clinical and imaging features of children with microcephaly or altered neuroimaging in the vertically exposed group to the Zika virus of the cohort from Goiania, Goias, Brazil, 2017-2019

| **Cases** | **Case 1** | **Case 2** | **Case 3** | **Case 4** | **Case 5** | **Case 6** |
| --- | --- | --- | --- | --- | --- | --- |
| Gestational age of maternal infection (weeks.days) | 18.0 | 36.2 | 12.5 | 12.0 | 11.6 | 27.2 |
| ZIKV exams (RT-PCR and/or serology); gestational age | RT-PCR: detected; 18.1 | RT-PCR: detected; 36.4 | RT-PCR: detected; 13.1 | RT-PCR: detected; 12.1 | IgM: reagent; 11.6 | IgM: reagent; 27.5 |
| Gestational age at birth (weeks.days) | 35.1 (preterm) | 40.4 | 37.6 | 38.3 | 39.5 | 38.1 |
| Sex | Male | Male | Male | Female | Male | Female |
| ZIKV exams (RT-PCR); infant’s age | - RT-PCR: detected;. | - RT-PCR: not done; | - RT-PCR: inconclusive; 2 days; - RT-PCR: not detected; 2 months; | - RT-PCR: not detected; 2 days;   . | - RT-PCR: not detected; 3 days; | - RT-PCR: not detected; 4 days); |
| Obstetric ultrasound | Intrauterine growth restriction, (IUGR) due to placental insuffiency | IUGR due to pre eclampsia | Reduction in head circumference ( microcephaly), periventricular calcifications, ventriculomegaly of 13 mm | Reduction in head circumference( microcephaly), periventricular calcifications, ventriculomegaly of 13 mm and IUGR | Suspected IUGR at 3^d^ trimester | Oligohydramnios with normal fetal dopller |
| Head circumference at birth, cm (Z score) | 28 (> -3 < -2) | 31 (> -3 < -2) | 30 (> -3 < -2) | 29 (< -3) | 34 (> -2 < -1) | 34 (> -1 < 0) |
| Birth weigth, grams (Z score) | 1,270 (> -3 < -2) | 2,480 (> -3 < -2) | 2,655 (> -2 < -1) | 2,650 (> -2 < -1) | 3,150 (> -1 < 0) | 2,820 (> -2 < -1) |
| Microcephaly at birth (MC) | Proportional MC | Proportional MC | Disproportionate MC | Disproportionate MC | No MC | No MC |
| Transfontanellar ultrasound (TF-US), age | Not done | Not done | Unilateral ventriculomegaly of 1.28 cm and MC, at birth | Bilateral ventriculomegaly, cerebral atrophy with enlargment of interhemispheric grooves, periventricular and basal ganglia calcifications, 23 days | 1^th^ TF-US - bilateral ventriculomegaly and fronto-parietal bilateral microcalcifications, 2 days  2^nd^ TF-US - compatible with right grade II intraventricular hemorrhage, 1 month | Few bilateral linear calcifications of talamic vessels, at birth |
| Post natal microcephaly | No MC | No MC | MC | MC | MC | No MC |
| Neuroimages: computed tomography (CT) and/or magnetic resonance image (MRI), age | Not done | Not done | CT: loss of brain volume, multiple calcifications, ventriculomegaly, 3 months | MRI: decreased brain volume, subependymal cysts, mega magna cistern, bilateral subcortical and periventrycular calcifications and basal ganglia, hypoplasia of the corpus callosum, 15 days | CT: multiple cortical and subcortical calcifications, right brain atrophy with simplification of gyri, right lateral ventricle dilation, 12 months | Not done |
| Eye abnormalities | Yes | Yes | Yes | No | Yes | Not done |
| Retinography | Optic nerve hypoplasia, optic nerv pallor, optic nerve excavation, chorioretinal atrophy, and focal pigmentary mottling | Chorioretinal atrophy | Vasculitis, chorioretinal scarring, and focal pigmentary mottling | Not done | Ectopic macula, hypopigmented macular lesion | Not done |
| Neurological abnormalities^a^ | No | No | Yes | Yes | Yes | Yes |
| Clinical signs |  | - | - At birth: overlapping sutures, extra skin on the neck and back, arthrogryposis. - 6 months: head circumference of 34 cm (Z score <-3), seizures, strabismus, and nystagmus. | - 57 days: severe neurological delay, central hypotonia, and spasticity of the 4 limbs. - 7 months: epilepsy, dysphagia, and irritability. | - 28 days: evolved with post natal moderate MC. - 8 months: severe microcephaly (head circumference=38 cm; Z score <-3), reduced segmental movements, altered archaic reflexes, mainly left deep reflexes abolished, hypertonia of the lower limbs, and hypotonia of the upper limbs. | - 12 months: delay in language, adaptive behavior and gross motor skills. |
| Medical evaluations | 2, 3, and 7 months | 8 months | 1, 6, and 11 months | Every 2 to 3 months | 28 days, 3, 8, and 12 months | 22 days, 3, and 12 months |
| Major and minor signs at follow up^a^ | - Minor signs: optic nerve hypoplasia, chorioretinal atrophy, and focal pigmentary mottling. | - Minor signs: chorioretinal atrophy. | - Major signs: ventricular enlargement >12 mm, multiple calcifications, epilepsy, and postpartum microcephaly (Z score <-3). - Minor signs: neonatal microcephaly (Z score <-2, IUGR, cognitive alteration, nystagmus, chorioretinal scarring, and focal pigmentary mottling. | - Major signs: ventriculomegaly >12 mm, multiple calcifications, pre and post natal MC, epilepsy, large cisterna magna, hypertonia, and seizures. - Minor signs: subependymal cysts, cognitive impairment, hyperexcitability, and nystagmus. | - Major signs: multiple microcalcifications, ventriculomegaly, postpartum microcephaly (Z score < -3), and lower limb hypertonia. - Minor signs: IUGR, hypotonia, partial immobility, cognitive disabilities, auditory alteration, and right optic nerve hypoplasia. | - Minor signs: oligohydramnios, and lenticulostriate vessels vasculopathy (calcifications), delay in neuropsychomotor development |
| CZS classificationᵃ | Mild/moderate signs potencially associated to ZIKV | Asymptomatic | Severe complications compatible with ZIKV | Severe complications compatible with ZIKV | Severe complications compatible with ZIKV | Mild/moderate signs potencially associated to ZIKV |

a. Pomar et al. (2018; 2019): asymptomatic was defined as no major signs and less than two minor signs; mild/moderate signs potentially associated with CZS were defined as no major signs and at least two ninor signs; severe complications compatible with CZS were defined as one major sign or three minor signs including at least one cerebral anomaly identified on prenatal or postnatal ultrasound.
