# Supplementary material for: Risk of adverse pregnancy and infant outcomes associated with prenatal Zika virus infection: a post-epidemic cohort in Central-West Brazil
Source: Sci Rep. 2023 May 5;13:7335. doi: 10.1038/s41598-023-33334-5 (PMC10161159; doi:10.1038/s41598-023-33334-5)
Supplement: Supplementary file 2 — Supplementary Information 2. [file 41598_2023_33334_MOESM2_ESM.docx]

Table 2 (Supplementary) – Ophthalmologic abnormalities probably related to vertical ZIKV exposure in children without microcephaly or altered brain imaging of the cohort from Goiania, Goias, Brazil, 2017-2019

| Children (n°) | **1** | **2** | **3** | **4** | **5** | **6** | **7** | **8** | **9** | **10** | **11** | **12** | **13** | **14** | **15** | **16** | **17** | **18** | **19** | **20** |
| --- | --- | --- | --- | --- | --- | --- | --- | --- | --- | --- | --- | --- | --- | --- | --- | --- | --- | --- | --- | --- |
| GA symptoms | 23w6d | 2w0d | 35w3d | 24w1d | 6w4d | 4w2d | 18w2d | 18w1d | 11w2d | 11w2d | 6w5d | 35w2d | 10w5d | 11w2d | 15w2d | 23w0d | 37w2d | 37w2d | 13w0d | 13w0d |
| Sex | Male | Male | Male | Female | Male | Female | Female | Male | Female | Male | Female | Female | Female | Female | Male | Male | Female | Male | Male | Female |
| Prematurity | N | N | N | N | 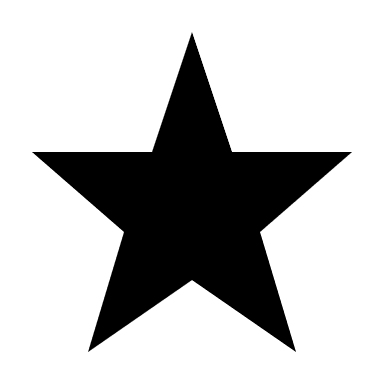 | N | N | N | 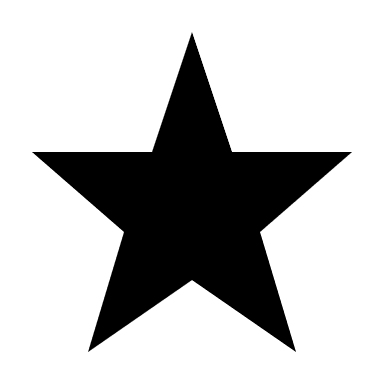 | 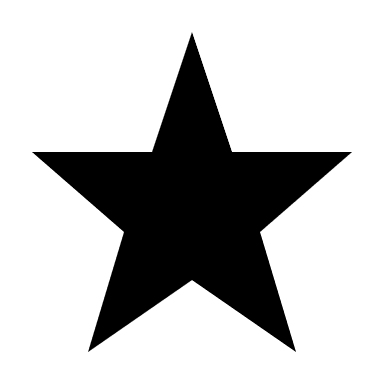 | N | N | 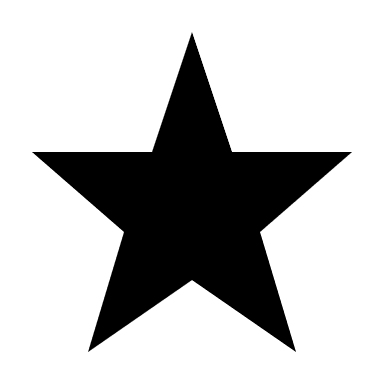 | N | N | N | N | N | 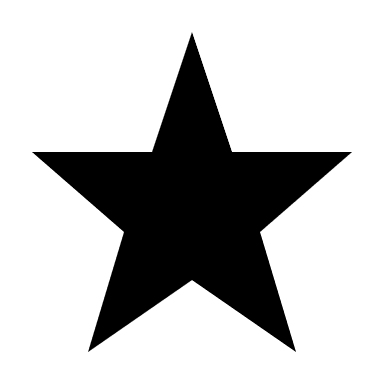 | N |
| IUGR | N | N | N | N | N | N | N | N | N | N | N | N | N | N | N | N | N | N | N | N |
| Diagnostic test* | R | R | R | F | R | R | F | R | R | R | R | F | R | F | R | R | R | R | R | R |
| Optic nerve anomaly | N | 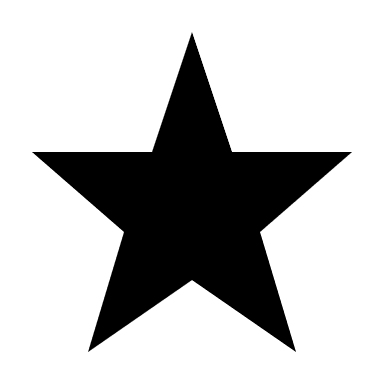 | N | N | 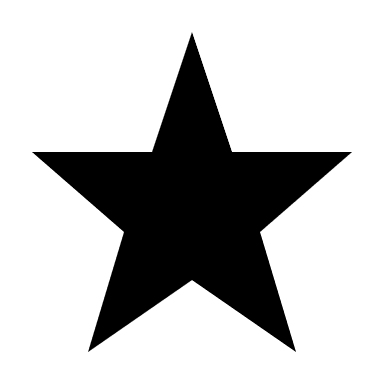 | N | O | N | N | N | 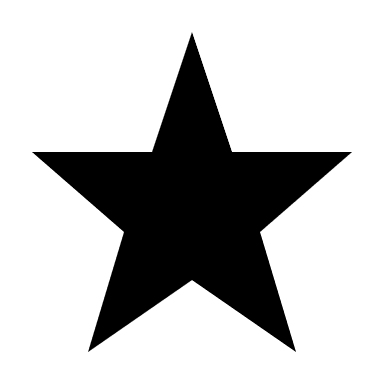 | N | N | N | N | N | N | 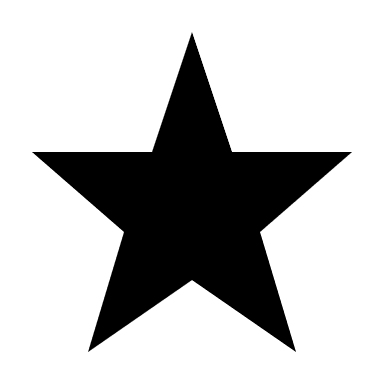 | N | N |
| Hypoplasia | N | 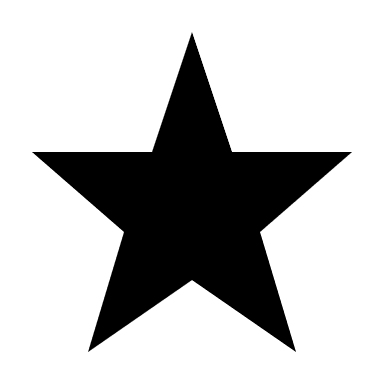 | N | N | 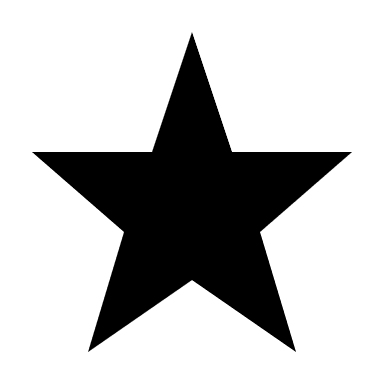 | N | N | N | N | N | 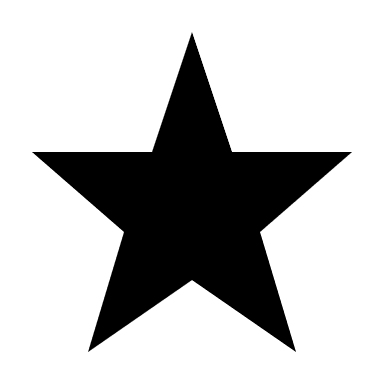 | N | N | N | N | N | N | 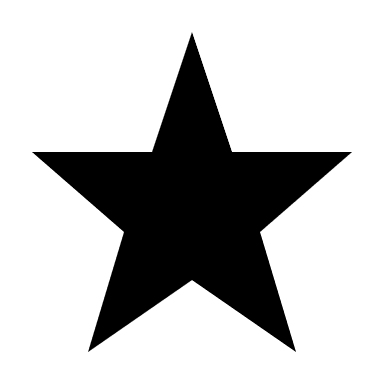 | N | N |
| Pallor | N | N | N | N | 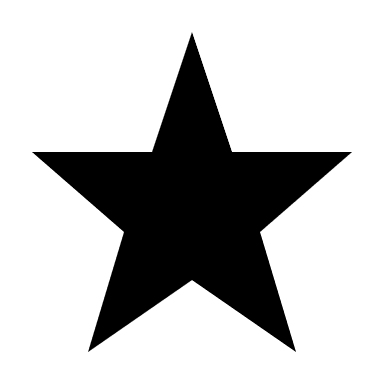 | N | N | N | N | N | 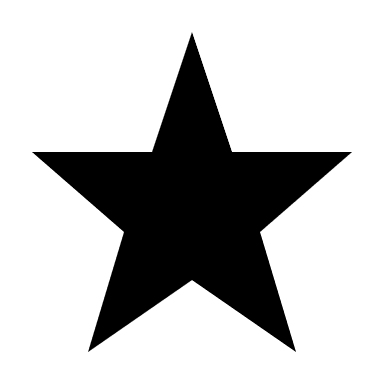 | N | N | N | N | N | N | N | N | N |
| Excavation | N | N | N | N | 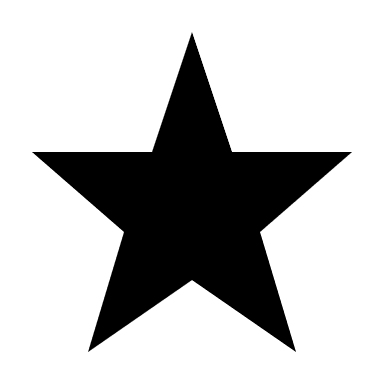 | N | 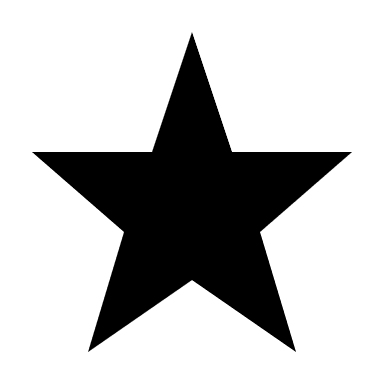 | N | N | N | 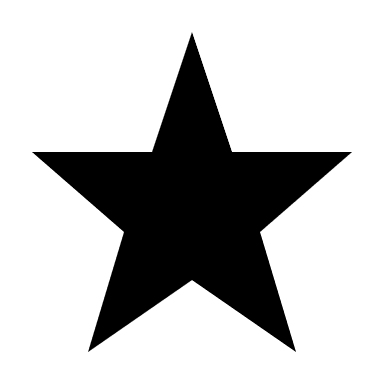 | N | N | N | N | N | N | N | N | N |
| Retinal anomaly | 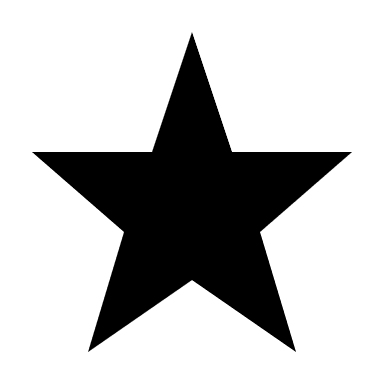 | 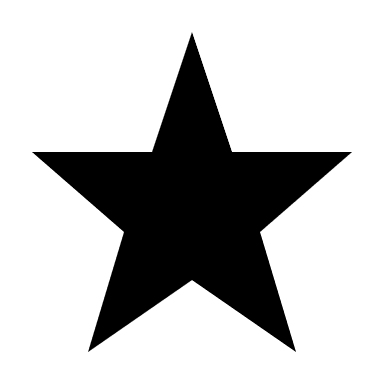 | 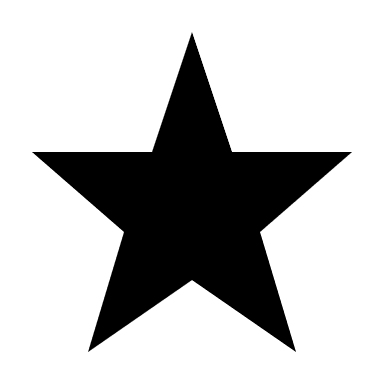 | 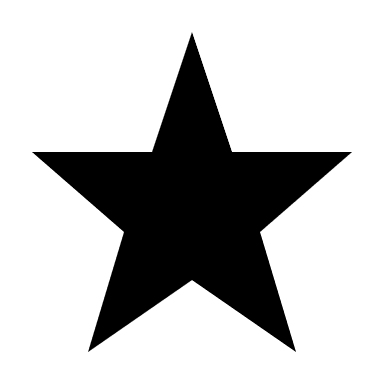 | 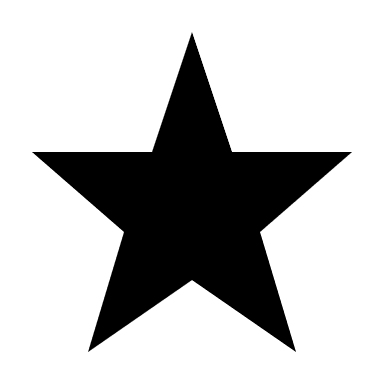 | 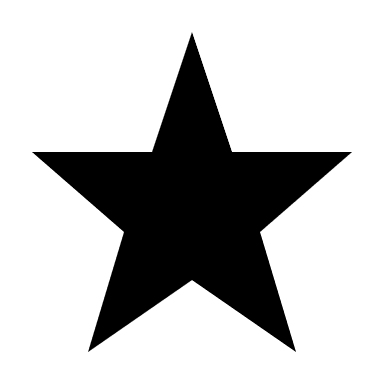 | 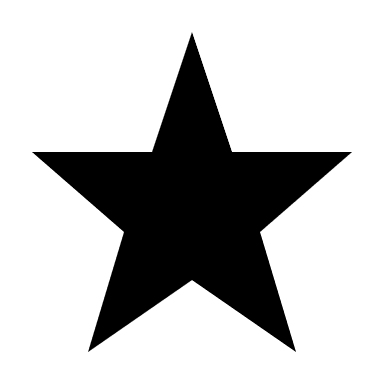 | 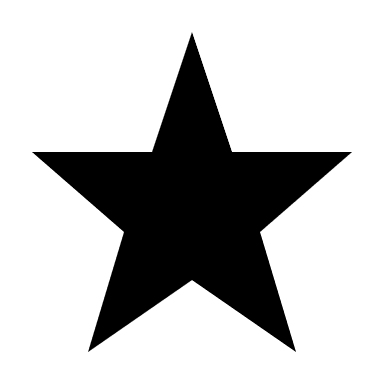 | 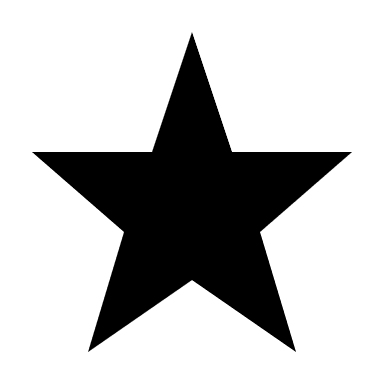 | 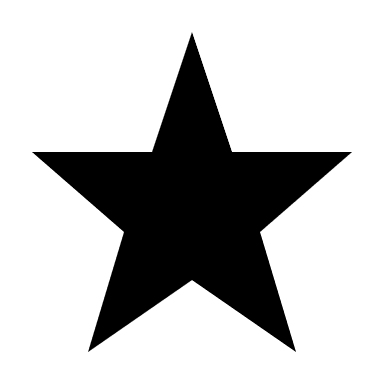 | 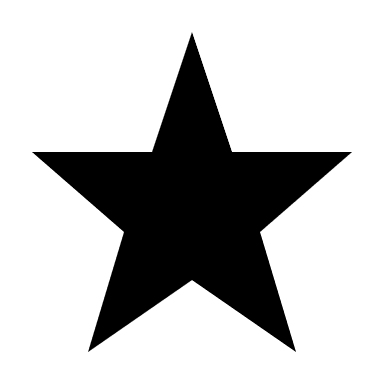 | 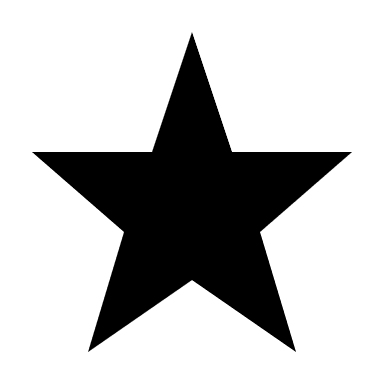 | 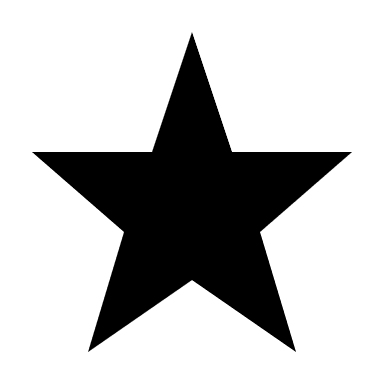 | 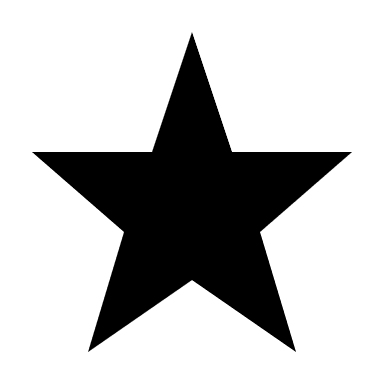 | 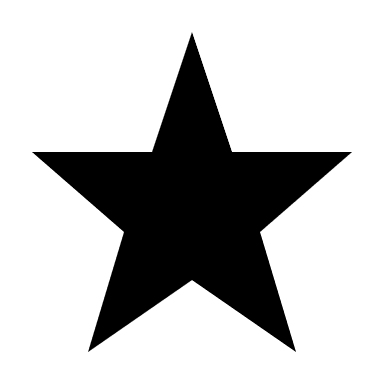 | 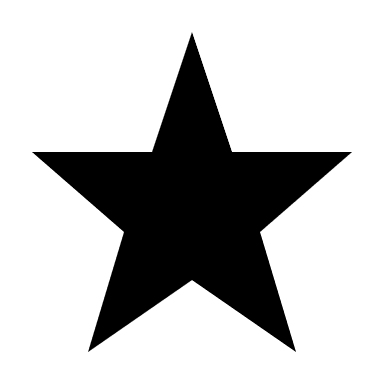 | 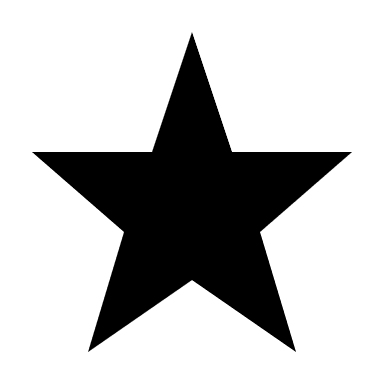 | N | 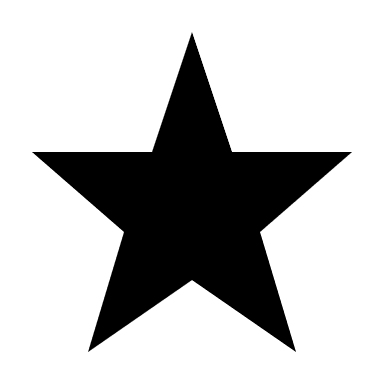 | 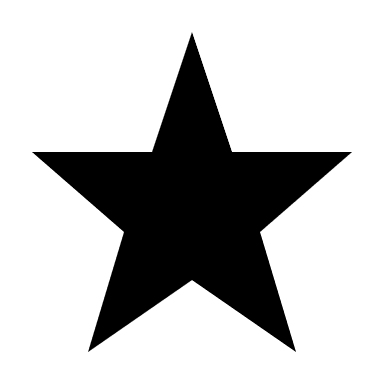 |
| Chorioretinal atrophy | 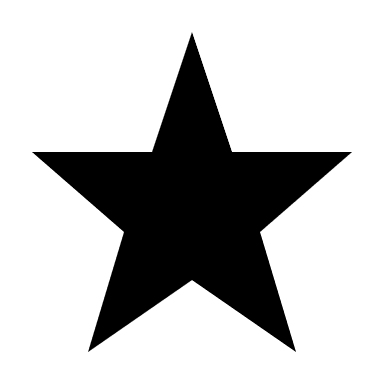 | N | N | 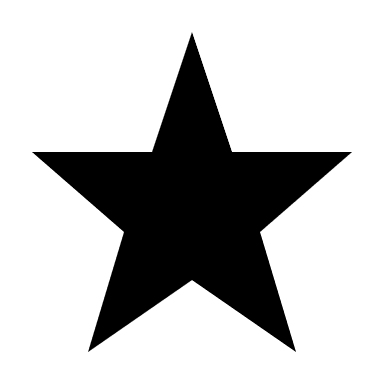 | N | N | N | 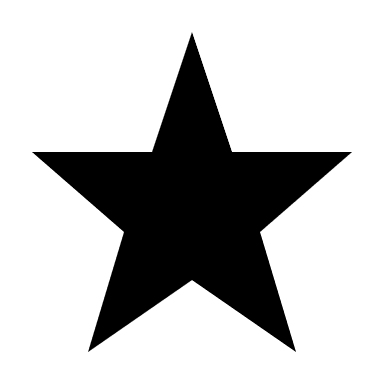 | N | N | N | N | 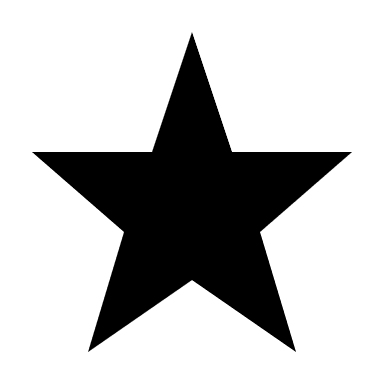 | N | N | N | N | N | 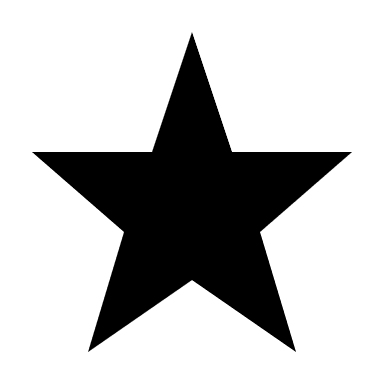 | 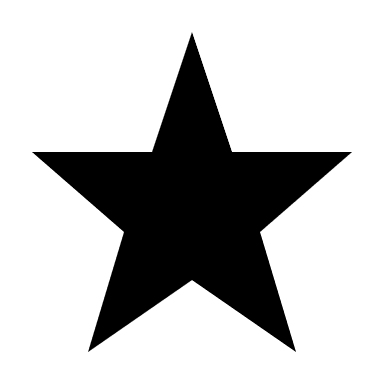 |
| Chorioretinal scarring | N | 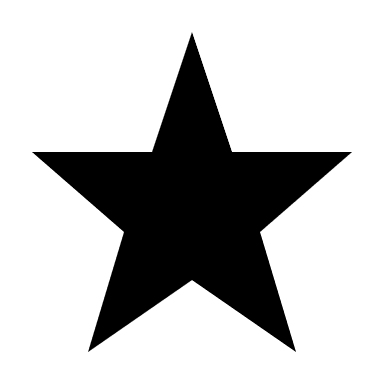 | N | N | N | N | N | N | N | 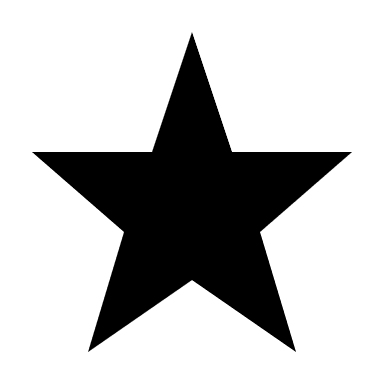 | 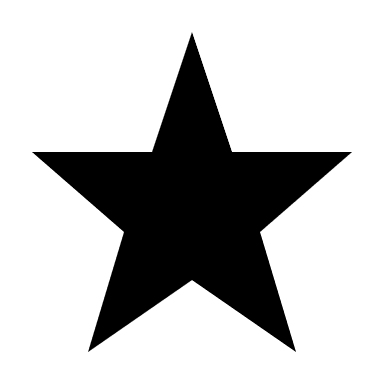 | N | N | N | N | N | N | N | N | N |
| Focal pigmentary mottling | N | N | 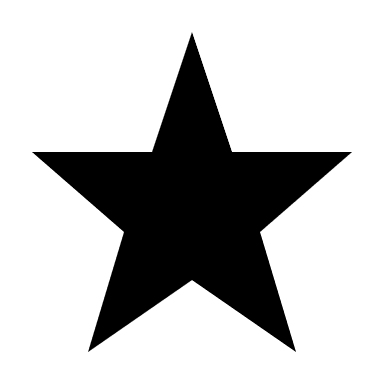 | N | 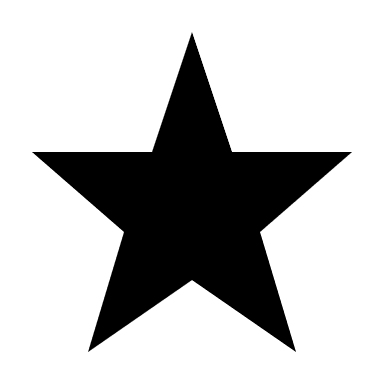 | N | N | N | 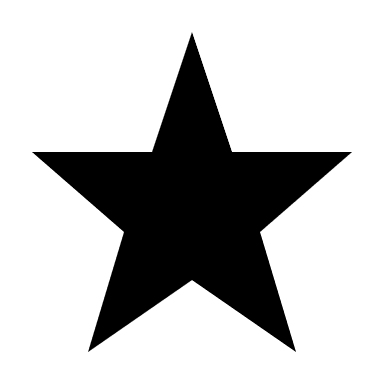 | 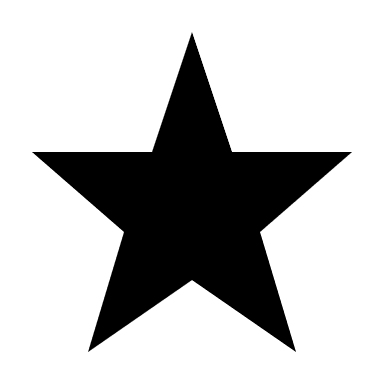 | 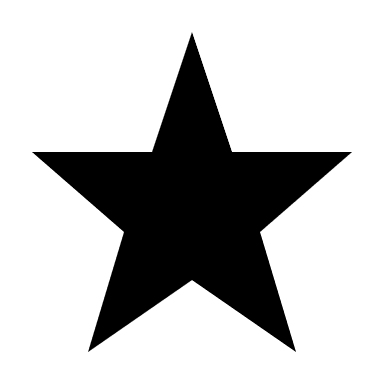 | 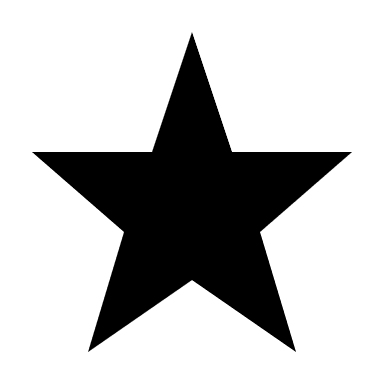 | 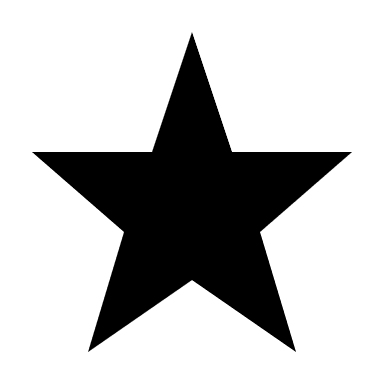 | 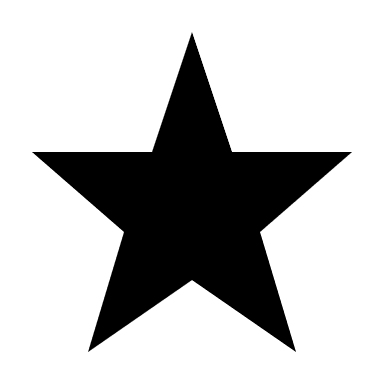 | N | 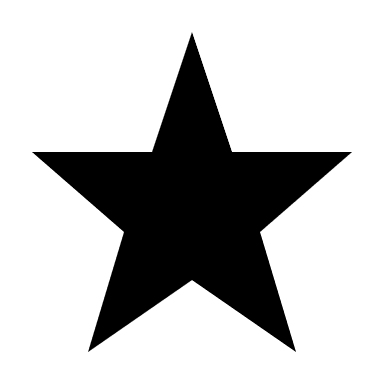 | 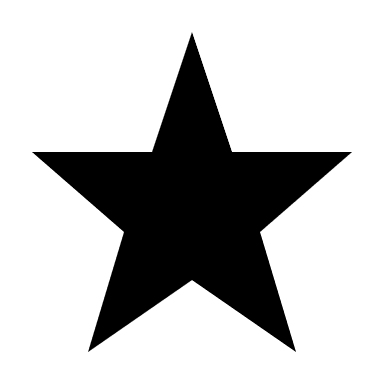 | N | 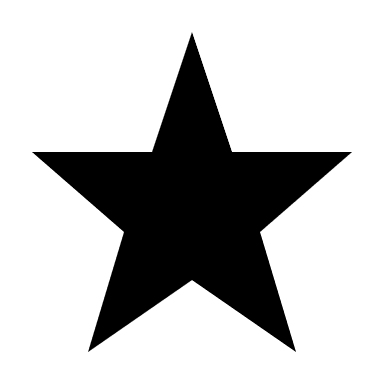 | 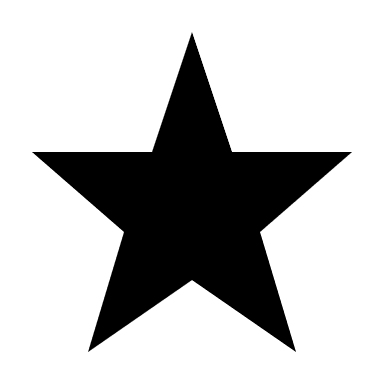 |
| Retinal hemorrhage | 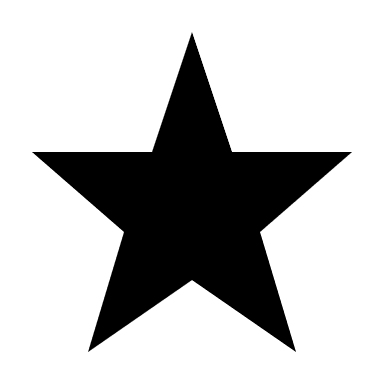 | N | N | N | N | N | N | N | N | N | N | 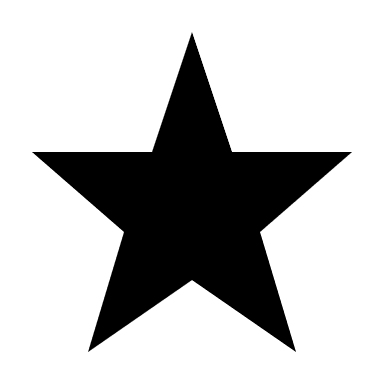 | N | N | N | N | 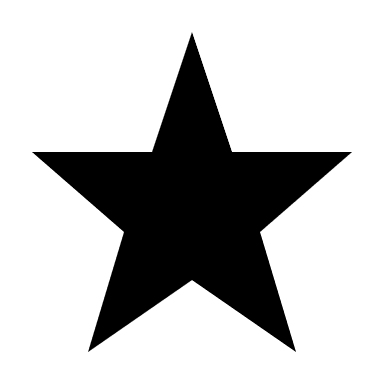 | N | N | N |
| Retinal vascular tortuosity | N | N | N | N | N | N | N | N | N | N | N | N | N | N | N | N | N | N | N | 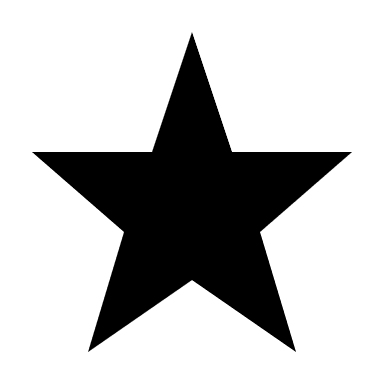 |
| Peripheral pigment dispersion | N | N | N | N | 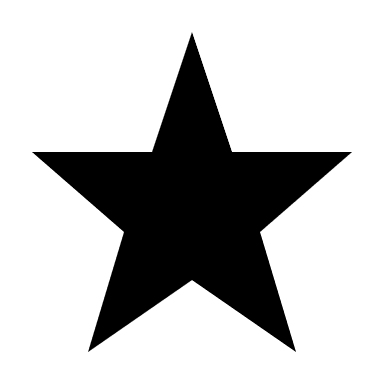 | 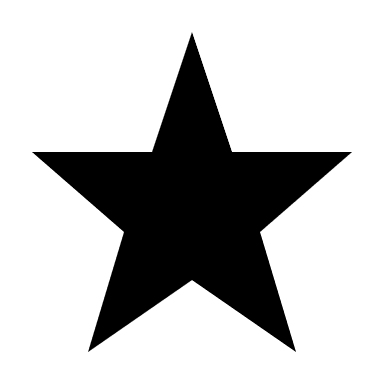 | 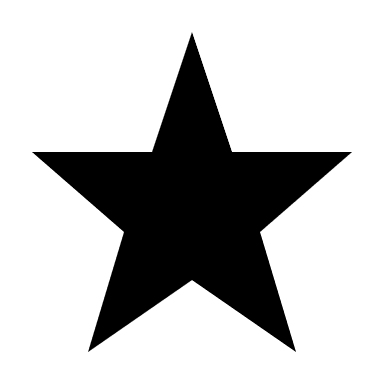 | 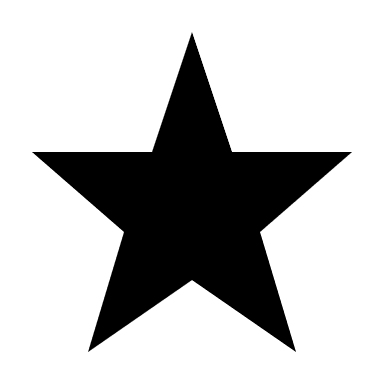 | N | N | N | N | N | N | 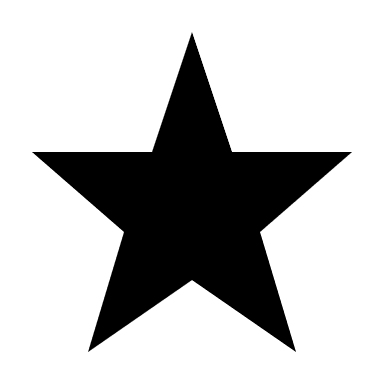 | N | N | N | N | N |
| Retinophaty of prematury | N | N | N | N | N | N | N | N | 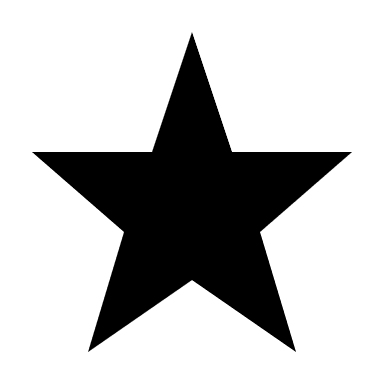 | 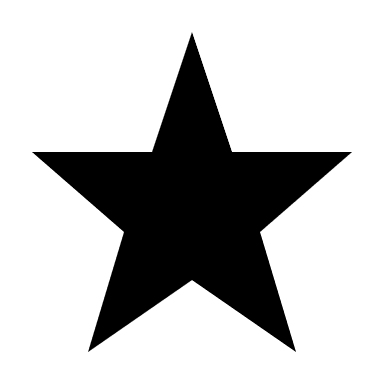 | N | N | 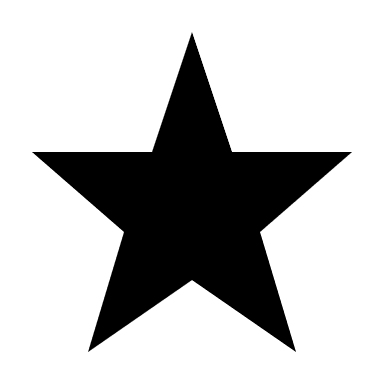 | N | N | N | N | N | N | N |

GA: gestational age. IUGR: intrauterine growth restriction. Diagnostic test*: all babies did fundoscopy. Ophthalmological abnormalities: N: no;
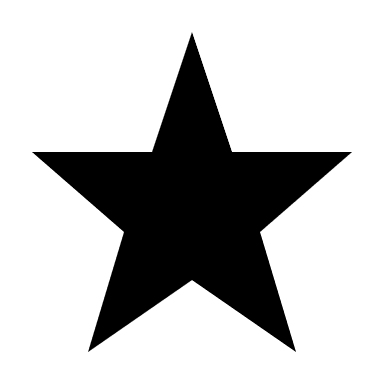
: yes; O: optic nerve anomaly not related to ZIKV. Diagnostic test: R: retinography; F: fundoscopy; R/F: retinography e fundoscopy.
